# Physiology

**Published:** 1879-04

**Authors:** 


### PHYSIOLOGY.

ON ALCOHOL.—M. Rabuteau. (*Progrès Médical*, December 28, 1878, p. 979.)

M. Rabuteau exhibited to the Society of Biology a specimen of alcohol obtained from potatoes. It consisted, according to M. B., of 50 per cent. of ethylic alcohol, 15 in 1,000 of isopropylic alcohol, traces of propylic alcohol and of two varieties of amylic alcohol. He has made experiments with these varieties of alcohol on frogs. He finds that a watery solution of ethylic alcohol, 1 in 50, produces no injurious effects on the frog; a solution of the same strength of isopropylic alcohol kills them in a few hours; a solution of same strength of propylic alcohol kills them in an hour, while a solution of the same strength of amylic alcohol kills them instantaneously. Amylic alcohol is in fact poisonous to frogs, he states, when used in the proportion of 1 pint to 500 parts of water. The frogs were subjected to the vapor of the solution. He insists on the differentiation of the affection known as alcoholism into two varieties: *Ethylism*, characterised by the laughing joviality of old Bacchus, and *Amylism*, or *Polyalcoholism*, which is characterised by the heavy stupidity arising from the modern whisky.

*Hygiene of the Eye.*—In the same session of the Society of Biology he presented the results of his researches on the well-being of the eye. He claims that if the number of near-sighted persons have increased in France, it is because sufficient attention has not been paid to the causes of the fatigue of that organ. Every one knows, he adds, that the eye soon becomes tired by looking at any article printed in squares of different colors. The same effect follows on reading from a book which remains fixed on the table, whereas if the book is held in the hands, and thus subject to a gentle motion, the fatigue is much less.

Black lines also on a white ground, such as are found in the majority of books, also fatigue the eye unnecessarily. A yellowish ground, he claims is the least fatiguing.

Another special cause of fatigue is reading of long lines.

In order to avoid the increase of myopia he suggests:

1. Let the light in the schools be abundant.
2. Let the type be large and on paper with a yellowish tint.
3. Lines of six centimeters in length are the least injurious.

